# A 3D-induced pluripotent stem cell-derived human neural culture model to study certain molecular and biochemical aspects of Alzheimer’s disease

**DOI:** 10.1007/s44164-022-00038-5

**Published:** 2022-11-14

**Authors:** Preeti Prasannan, Elodie Siney, Shreyasi Chatterjee, David Johnston, Mohammad Shah, Amrit Mudher, Sandrine Willaime-Morawek

**Affiliations:** 1 Faculty of Medicine, University of Southampton, Southampton, UK; 2 School of Biological Sciences, University of Southampton, Southampton, UK

**Keywords:** 3D cell cultures, Induced pluripotent stem cells, Stem cell differentiation, Alzheimer’s disease

## Abstract

**Purpose:**

Alzheimer’s disease (AD) early pathology needs better understanding and models. Here, we describe a human induced pluripotent stem cells (iPSCs)-derived 3D neural culture model to study certain aspects of AD biochemistry and pathology.

**Method:**

iPSCs derived from controls and AD patients with Presenilin1 mutations were cultured in a 3D platform with a similar microenvironment to the brain, to differentiate into neurons and astrocytes and self-organise into 3D structures by 3 weeks of differentiation in vitro.

**Results:**

Cells express astrocytic (GFAP), neuronal (β3-Tubulin, MAP2), glutamatergic (VGLUT1), GABAergic (GAD65/67), pre-synaptic (Synapsin1) markers and a low level of neural progenitor cell (Nestin) marker after 6 and 12 weeks of differentiation in 3D. The foetal 3R Tau isoforms and adult 4R Tau isoforms were detected at 6 weeks post differentiation, showing advanced neuronal maturity. In the 3D AD cells, total and insoluble Tau levels were higher than in 3D control cells.

**Conclusion:**

Our data indicates that this model may recapitulate the early biochemical and pathological disease features and can be a relevant platform for studying early cellular and biochemical changes and the identification of drug targets.

**Supplementary Information:**

The online version contains supplementary material available at 10.1007/s44164-022-00038-5.

## Introduction

Alzheimer’s disease (AD) is an irreversible age-related progressive neurocognitive disorder and the most common cause of dementia. In the UK, one in 14 people over 65 years of age have dementia [1]. In 2019, a study showed that 885,000 people are estimated to be living with dementia in the UK and this number is expected to increase exponentially over the next few decades [2]. People suffering from AD show severe memory impairment which is attributed to two primary hallmarks of the disease: amyloid plaques and neurofibrillary tangles. Plaques are extracellular deposits of the protein β-amyloid (Aβ) and tangles are intracellular deposits of the microtubule-associated protein Tau [3–6]. The early onset familial disease is attributed to the patients carrying a mutation in amyloid precursor protein (APP), Presenilin 1 (PSEN1) and Presenilin 2 (PSEN2) genes, with most mutations present in PSEN1 [7–10]. PSEN1 and PSEN2 genes encode the respective PS-1 and PS-2 catalytic subunits of the enzyme complex γ-secretase [11–13]. While familial AD (FAD) cases account for less than 5% of the AD cases, sporadic AD (SAD) is the most common form of AD [14, 15]. Despite SAD being the most prevalent form of AD, most of our understanding of the underlying pathology of the disease comes from studies on FAD models. While studies in post-mortem human brain tissue and transgenic models over-expressing FAD mutations have provided some insights about the underlying mechanisms of the disease, there is a move towards human-cell-based models in recent years. In the leap from bench to bedside, human-based-physiologically relevant *in vitro* models are key in understanding and overcoming the evolutionary and special differences of the underlying disease mechanisms which are poorly represented by animal models [16]. Induced pluripotent stem cell (iPSC) technology allows for the unlimited generation of stem cells with the ability to differentiate into neurons and astrocytes directly from easily available skin or blood cells of patients with specific mutations[17–19]. This method ensures the expression of underlying pathology without forcing overexpression of genes/mutations of interest.

However, despite the advantages of the iPSC technology, the development of disease models that simulate protein aggregation using conventional 2D culture techniques remains challenging. Although conventional 2D cultures have been quick and cheap tools for obtaining valuable disease-relevant insight, they are cultures grown on rigid and flat artificial substrates such as glass or polystyrene which poorly represent the *in vivo* physiological microenvironment. 2D culture systems do not mimick the complexity of the human brain, thereby misrepresenting morphology, differentiation, synaptic architecture especially cell–cell and cell-microenvironment interactions. The advent of 3D tissue engineering has revolutionised the approach towards the conventional *in vitro* culture systems. Studies have shown 3D cultures not only encourage physiologically relevant morphology/phenotype of different cell types, accelerate differentiation and formation of intrinsic neural networks but also recapitulate the key pathological features of the disease *in vitro* which were never reported before in 2D culture systems [20–25]. Hence, differentiating patient-derived iPSCs to generate disease-relevant cell phenotypes would help overcome most of the issues seen in *in vitro* models.

Most of the disease-modifying drugs have failed in clinical trials due to the administration in the late phase of the disease, hence, there is good reason to study and understand the early changes seen in the pathogenesis. Until recently, it was believed that the accumulation of Aβ was the point of initiation of the disease in the brain, which eventually led to the formation of plaques and hyperphosphorylated neurofibrillary tangle [4, 26, 27]. In recent years, the field has moved to appreciating a casual role of the Tau protein with many Tau-centric drugs targeting Tau mechanisms of toxicity [28]. Although mutation of the gene encoding for Tau is not directly implicated in AD, the wild-type protein becomes abnormally hyperphosphorylated and aggregated in the disease [29] in both FAD and SAD cases. However, the mechanism initiating the process is unknown. A number of studies using either human neurons overexpressing specific FAD genes or AD patients iPSC derived neurons have described increased levels of phosphorylated Tau and increased insolubility of the protein [25, 30, 31]. Some have reported increased Tau protein levels in iPSC models with APP mutations [32, 33]. A key question is whether these Tau phenotypes are specific to cells with APP mutations or also seen in cells with PSEN1 mutations and whether iPSC models can be used to establish links between Aβ and Tau-associated changes in the disease.

Here, we report a human *in vitro* model to study such early changes seen during the development of the pathology. We report the differentiation of human iPSC-derived neural stem cells (hiNSCs) into neurons and astrocytes in 3D cultures over a period of 12 weeks *in vitro.* We also report that the patient-derived hiNSC with PSEN1 mutations show higher levels of total and insoluble Tau *in vitro*. Our findings support that our 3D cultures are a relevant model to study certain molecular and biochemical pathological aspects of AD, especially during the early phase of the disease.

## Methods

### iPSC-derived neural stem cell lines and culture reagents

**Fig. 1:**
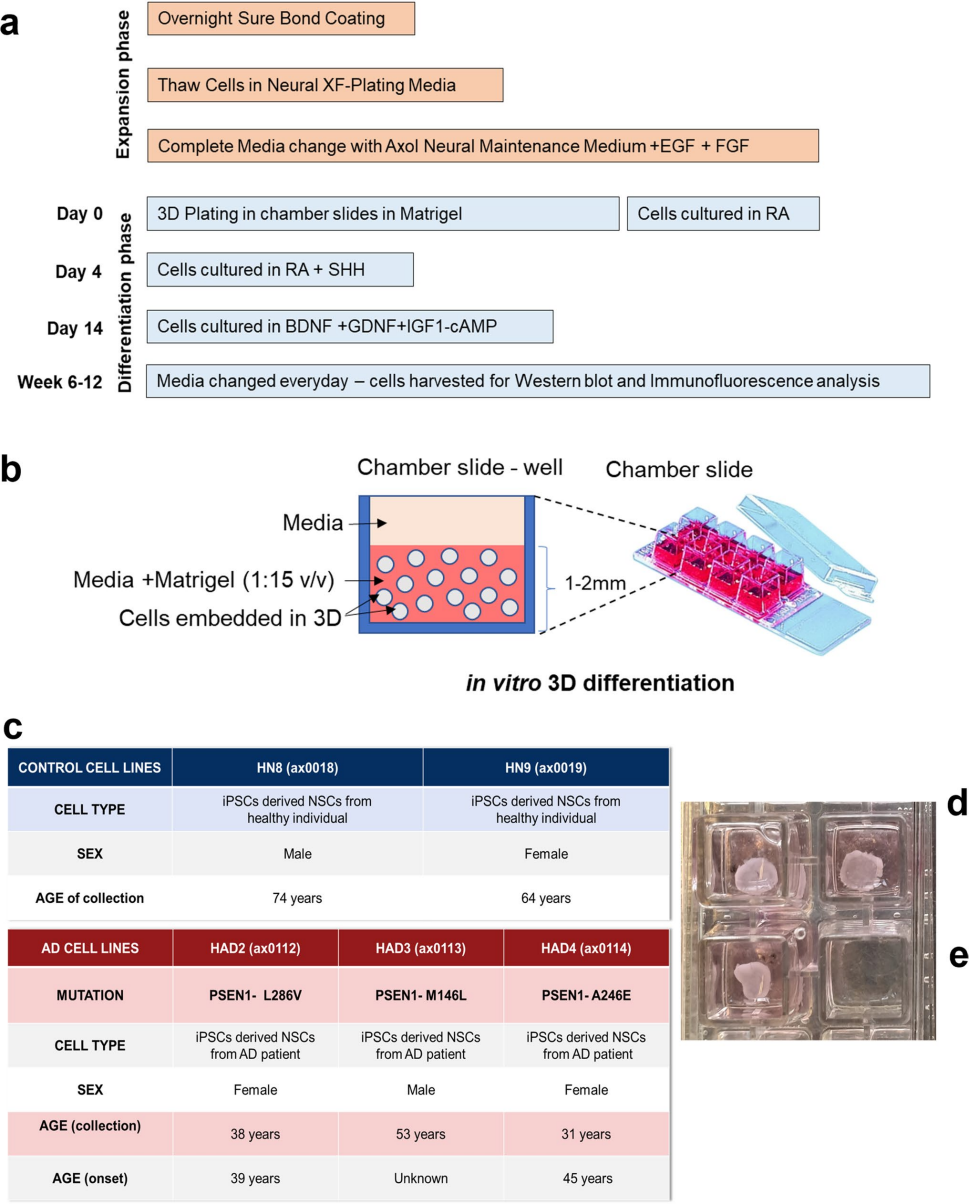
Generation of human iPSC-derived 3D neural cultures.** a** Details of the method followed to culture cells in 3D. RA, retinoic acid; SHH, Sonic Hedgehog; BDNF, Brain-Derived Neurotrophic growth Factor; GDNF, Glial cell line Derived Neurotrophic Factor; IGF1, Insulin-like Growth Factor 1; and cAMP, cyclic Adenosine Monophosphate.* b* Schematic diagram representing the *in vitro* 3D culture model. **c** Details of the iPSC-derived NSCs (control and AD patient-derived) from Axol Biosciences. **d** Image of 3D cultures in chamber slides at 5 weeks post differentiation showing the cells have self-organised to create a sheet which has detached from the sides of the culture walls and is free-floating. **e **Image of 3D cultures in chamber slides at 6 weeks post differentiation showing the free-floating sheet has curled up and folded itself into a ball-like structure

Human iPSC-derived neural stem cells (hiNSCs) were purchased from Axol Biosciences Ltd. (Axol Biosciences, Cambridge, UK). AD-hiNSCs lines were derived from AD patients with PSEN 1 mutations-L286V (ax0112, Axol Biosciences, UK) M146L (ax0113, Axol Biosciences, UK) and A246E (ax0114, Axol Biosciences, UK). Age-matched control-hiNSCs lines (HN8, ax0018, Axol Biosciences, UK and HN9 ax0019) were derived from healthy individuals (Fig. 1). Skin fibroblasts were used to generate the hiPSCs by using integration-free episomes and hiNSCs were derived under fully defined neural induction conditions. The hiNSCs were maintained in Axol Neural Maintenance (ANM) media composed of Axol Neural Basal Media (ANB media-ax0013b, Axol Biosciences, Cambridge, UK) supplemented with 1.5% (v/v) Axol Neural Supplement (ANS-ax0031a, Axol Biosciences, Cambridge, UK) and 1% (v/v) Antibiotic–Antimycotic solution (A5955, Sigma-Aldrich, UK).

### Thawing and expansion of iPSC-derived neural stem cells

SureBond XF (1000x, ax0041XF, Axol Biosciences, Cambridge, UK) was slowly defrosted at 4 °C 2 days prior to thawing cells, to prevent jellification. SureBond XF was diluted (5 µl/ml) with sterile PBS without Ca-Mg (10010023, Gibco, UK) to coat T75 culture flasks one day prior to plating cells. Cells were defrosted, transferred to prewarmed fresh ANM media and centrifuged at 200 g for 5 min. Cells were then transferred to fresh pre-warmed neural plating—XF Media (ax0033, Axol Bioscience, UK). Cells were left to stabilise for 24 h in the incubators in this media. The next day, the media was completely replaced with fresh pre-warmed ANM media supplemented with EGF (0.1 mg/ml, AF-10015, PeproTech, UK) and FGF (0.05 mg/ml, 100-18B, PeproTech, UK). Media was changed every second day for 6 days. Cells lines were not passaged more than once during the expansion phase. The same differentiation protocol was followed for both the 2D and 3D cell culture methods.

### 3D cell culture plating

For 3D seeding, cells were washed with PBS. Warm Axol Unlock XF Media (ax0044XF, Axol Biosciences, UK) was added to the cells for 3 min at 37 °C for the cells to detach. Cells were recovered in ANM media and spun at 200 g for 5 min. The pellet was resuspended in fresh warm ANM media. Cells were plated on ice, in an 8-well chamber slide (177445, Lab-Tek system, Nunc, UK) at a density of 2 × 10^6^/ml in 3D plating media composed of ANM media with Retinoic Acid (RA-R2625, Sigma-Aldrich, UK) at 500 ng/ml and supplemented with Matrigel (354230, Corning) at a ratio of 1:15 v/v, and then left to form a gel at 37 °C. An extra 200 µl of ANM media was added 2 h after the cultures were plated.

### 2D cell culture plating

For 2D plating, cells from the flasks were washed with PBS and detached using warm Unlock XF Media (ax0044XF, Axol Biosciences, UK) for 3 min at 37 °C. Detached cells were then diluted in 15 ml of ANM media and centrifuged at 200 g for 5 min. The pellet was resuspended in fresh warm ANM media. For plating cells in 2D, 5-mm-diameter/1.5-mm-depth round glass coverslips were coated with 0.01% solution of Poly-L-Ornithine (P 495, Sigma-Aldrich, UK) overnight followed by coating with 50 µg/ml Laminin (L2020, Sigma-Aldrich, UK) for 1 h at 37 °C. 6000 cells were seeded evenly per well in a 6-well dish with coated coverslips and placed at 37 °C. Cells were allowed to attach for 2 h before supplementing with 500 ng/ml RA.

### Differentiation of iPSC-derived NSCs in 2D and 3D cultures

Four days after seeding cells for 2D or 3D cultures, the cells were supplemented with Sonic Hedgehog (SHH, 100–45, PeproTech, UK) at 100 ng/ml. Two weeks after the initial cultures, the media was replaced with ANM media supplemented with a cocktail of growth factors: 0.01 mg/ml Brain-Derived Neurotrophic Factor (BDNF, 450–02, PeproTech, UK), 0.01 mg/ml Glial cell line Derived Neurotrophic factor (GDNF, 450–10, PeproTech, UK), 1 mg/ml cyclic Adenosine Monophosphate (cAMP, A9501 Sigma-Aldrich, UK), 0.01 mg/ml Insulin-like Growth Factor (IGF,1–100-11B, PeproTech, UK) to promote neural cell differentiation for 12 weeks. Media was changed every other day for the first 3 weeks followed by every day up to 12 weeks *in vitro*.

### Cryosectioning of 3D cultures

For embedding, the 3D cultures were fixed with 4% paraformaldehyde for 24 h at 4 °C. The cultures were then washed three times with PBS and cryoprotected in 30% sucrose solution (S9378, Sigma-Aldrich, UK) prior to embedding in optimal cutting temperature (O.C.T.) embedding medium (12678086, Thermo Scientific, UK) over dry ice. Frozen tissue was cryosectioned at 14 µm thickness using a S35 microtome blade (JDA-0100-0A, Cell Path, UK). Each section was collected and transferred onto SuperFrost glass micro slides (10149870, Fisher-Thermo Scientific, UK). Sections were stored at − 20 °C after being air dried at room temperature overnight.

### Immunofluorescence staining

For immunostaining of 2D cultures, the cells on the coverslips were fixed with 4% paraformaldehyde for 15 min at room temperature. To compare the two different systems of cultures, 2D coverslips and 3D culture sections were stained following the same protocol for each marker.

For immunolabelling with MAP2 (1:200, 4542, Cell Signalling), Nestin (1:200, MAB5326, Millipore) and Synapsin1 (1:200, ab8, Abcam); cells were permeabilized for 20 min in PBS (P4417, Sigma-Aldrich, UK) containing 0.2%Triton-X 100 (X100, Sigma-Aldrich, UK) (PBST) and then blocked in a solution containing 10% v/v donkey serum (S-30 M, Sigma-Aldrich, UK) in PBST for 1 h at room temperature followed by incubation with primary antibodies overnight at 4 °C in the blocking solution. Cells were washed thrice in PBST for 5 min each. The secondary antibodies used were species-specific 488 Alexa Fluor (1:200, A-21206, Invitrogen, UK) and 568 Alexa Fluor (1:200, A10037, Invitrogen, UK). Secondary antibodies were prepared in blocking solution and cells were incubated for 2 h at room temperature. Nuclear stain DAPI (D9542, Sigma-Aldrich, UK) was used at 2 µg/ml in permeabilization buffer for 15 min at room temperature.

For immunolabelling with β3-Tubulin (1:200, 801202, Biolegend), GFAP (1:200, ZO334, Dako Cytomation), VGLUT 1 (1:200, 135303, Synaptic Systems) and GAD65/67 (1:200, G5163, Merck); cells were permeabilized and blocked for 1.5 h in PBS (Sigma-Aldrich, UK) containing 1%Triton-X 100 (X100,Sigma-Aldrich, UK), 4% v/v donkey serum (S30-M, Sigma-Aldrich, U), 1% v/v Bovine Serum Albumin (BSA) (A9647, Merck, UK) and 0.3 M Glycine (BP381-1, Fisher Bioreagents, UK). Cells were then incubated with primary antibodies overnight at 4 °C in the same solution. Cells were washed thrice in PBS 1%Triton-X 100 for 5 min each. The secondary antibodies used were species-specific 488 Alexa Fluor (1:200, A-21206, Invitrogen, UK) and 568 Alexa Fluor (1:200, A10037, Invitrogen, UK). Secondary antibodies were prepared in the same blocking solution and cells were incubated for 2 h at room temperature. Nuclear stain DAPI (D9542, Sigma-Aldrich, UK) was used at 2 µg/ml in permeabilization buffer for 15 min at room temperature.

Slides were mounted and sealed with an in house-prepared, setting mountant comprising PBS, glycerol, Mowiol 4–88 (Harco) and Citifluor AF3 anti-fade solution (Citifluor Electron Microscopy Sciences. To decrease variability, all control and AD cells were stained and processed in parallel.

### Image analysis

Cells were imaged using a Leica TCS-SP8 laser scanning confocal microscope on a Leica DMi8 inverted microscope frame (TCS SP8, Leica Microsystems) at × 63 magnification using a glycerol immersion objective (Leica HC PL APO CS2, 1.3NA). Images were acquired at a 1024 × 1024 sample rate and 0.75 zoom (field of view—246 × 246 um, equivalent pixel size = 240 × 240 nm in XY). Maximum confocal detection sensitivity was set for each channel using control samples (secondary antibodies and DAPI only). Sequential imaging and optimised setting of detection bandwidths on the Leica TCS spectral detection system (Alexa Fluor 488 = 500–550 nm bandwidth, Alexa Fluor 568 = 575–650 nm bandwidth) were used to avoid crosstalk between channels. Fields of view to image were selected by conventional fluorescence microscopy using DAPI signal so as not to bias field selection based on the specific probe signal. For both 2D and 3D cultures, Z stacks were acquired at 1 µm Z intervals. For each 3D culture, cryosections from three independent cultures were imaged and three to five different fields were captured per section. For 2D cultures, three coverslips from three independent cultures were imaged and three to five fields were captured per coverslip. To compare between 3 and 2D cultures a representative single Z stack was used. Image analysis was performed using ImageJ software (Fiji). The threshold was determined manually for each image for each channel. For analysis, the entire field of view was analysed to measure the total area positive above the threshold for each channel to calculate the relative proportion of positive pixels. Each measured positive area was normalised against the amount of DAPI signal to normalise for variation in cell numbers between samples/fields.

### Lysate preparation for western blot analysis

Whole protein extracts were analysed for Tau protein and PHF1 in AD and control 3D cultures. The 3D cultures were homogenised in tris buffered saline (TBS) extraction buffer containing 50 mM Tris–HCl-pH 7.4, 175 mM NaCl, halts protease inhibitor cocktail (78,430, Thermo Fisher Scientific, UK), 0.1 mM Phenylmethylsulfonyl fluoride (PMSF, P20270, Thermo Fisher Scientific, UK) was added along with a mixture of phosphatase inhibitors (P52102-1, Melford, UK) at 4 °C.

### Sequential extraction of soluble and insoluble protein fractions for western blot analysis

Three sequential fractions, S1, S2 and S3, were extracted from each 3D sample processed (1 culture well) to study the differential solubility of Tau in the 3D cultures [34, 35]. Halts Protease Inhibitor Cocktail (78430, Thermo Fisher Scientific, UK), 0.1 mM Phenylmethylsulfonyl fluoride (PMSF, P20270, Thermo Fisher Scientific, UK) was added along with a mixture of phosphatase inhibitors (P52102-1, Melford, UK) to each of the three buffers. Three individual 3D cultures (for each cell line) at 6 weeks post differentiation, were homogenised at 4 °C in TBS extraction buffer containing 50 mM Tris–HCl (pH 7.4), 175 mM NaCl along with the aforementioned cocktail of protease and phosphatase inhibitors in 1.5 ml ultracentrifuge Eppendorf tubes (357448, Beckman Coulter, UK) with a plastic pestle. 15 µg of each lysate was run in a gel to be stained with the housekeeping protein β-Actin. The lysate was then ultra-centrifuged at 186,000 g for 2 h at 4 °C. The supernatant (S1-TBS soluble fraction) was collected and stored at − 80 °C. The insoluble pellet was resuspended in 5% SDS extraction buffer containing 50 mM Tris–HCl (pH 7.4), 175 mM NaCl and 5% SDS along with the inhibitors and was homogenised. The lysate was then ultra-centrifuged at 186,000 g for 2 h at 25 °C. The supernatant (S2-SDS soluble fraction) was stored at − 80 °C. The subsequent insoluble pellet was resuspended in 100 μl SDS extraction buffer and ultra-centrifuged for 1 h at 186,000 g at 25 °C. The pellet was rehomogenised in the UREA extraction buffer and agitated for 12–18 h at room temperature. The S3-SDS insoluble urea soluble lysate was stored at − 80 °C. Before loading into gels, all fractions were mixed with equal volumes of 2 × loading buffer (LB) and boiled for 5 min at 95 °C. Equal volumes of each fraction were loaded in the gel for western blotting. A semiquantitative method was used to measure the relative proportion of each fraction to total Tau, where total Tau is the cumulative measure of each fraction (normalised) of a cell line (Total Tau = S1 + S2 + S3).

### Western blot analysis

A total of 15 µg protein was resolved on 10% polyacrylamide gels at 120 V. The proteins were then transferred on to a nitrocellulose membrane (GE10600002, Merck-Sigma, UK) at 60 V for 90 min. The blots were blocked in TBS containing 0.5% Tween-20 (P9416, Merck-sigma, UK) and 5% BSA (A2153, Sigma-Aldrich, UK). Blots were incubated overnight with primary antibodies (Dako Tau (1:10,000, A0024, Dako), PHF1 (1:1,000, Gift from Peter Davis), RD3 (1:1,000, 05–803, Millipore), RD4 (1:500, 05–804 Millipore) and β-Actin (1:2,000, ab8224, Abcam)) at 4 °C on a shaker after which they were washed thrice in TBS 0.5% tween-20 for 5 min each. The blots were incubated with species-specific secondary antibodies (IRDye800CW (1:20,000, 926–32211, LI-COR) and IRDye680RD (1:20,000, 926–68070, LI-COR) for 1 h at room temperature. The blots were washed thrice for 5 min each with wash buffer. All blots were scanned using the Licor imaging system with Odyssey software. Image J (Fiji) was used to determine the intensity of the bands.

### Statistics

All values are presented as the mean ± standard error of the mean. To compare differences between the groups, statistical analysis was performed using GraphPad Prism version 8.4 (GraphPad software, Inc). One-way Anova was used for statistical analysis with Bonferroni’s multiple comparison post-hoc test for comparison across groups (with normally distributed data). For non-parametric values, the Kruskal–Wallis test was performed. For comparison between two groups (control lines vs AD, or 6 weeks vs 12 weeks) a two-tailed unpaired Student’s *t*-test was performed. For normally distributed data Welch’s correction was used, whereas for non-parametric data Mann–Whitney test was performed.

## Results

### Generation of iPSC-derived 3D cultures-3D Culture Setup

We hypothesised that the use of a 3D culture system would accelerate, as well as facilitate the development and maturation of neurons and astrocytes *in vitro*. We have developed a differentiation protocol to generate cortical neurons and astrocytes *in vitro* (Fig. 1a). We developed a scaffold-free system using Matrigel as the matrix to embed and support the differentiation of neurons and astrocytes since Matrigel has a high level of extracellular matrix proteins typically found in the brain (Fig. 1b). In order to investigate certain molecular and biochemical aspects of AD, we used iPSCs derived from AD patients with a PSEN1 mutation and from healthy individuals (Fig. 1c). Cells were grown in 3D for up to 12 weeks, immunohistochemistry and western blot were used to characterise the cultures. Cells initially appear spherical post-seeding in 3D cultures and eventually most grow out to form extensions. By 3–4 weeks, cells form extended structures and a densely interconnected network. By 5–6 weeks, cells aggregate together forming a self-organised sheet of cells, detaching from the walls of the culture well (Fig. 1d). By 6–7 weeks, these cell sheets detach completely from the well and curl up inwards to form free-floating ball-like structures (Fig. 1e).

### 3D cultures contain astrocytes, glutamatergic and GABAergic mature neurons

**Fig. 2:**
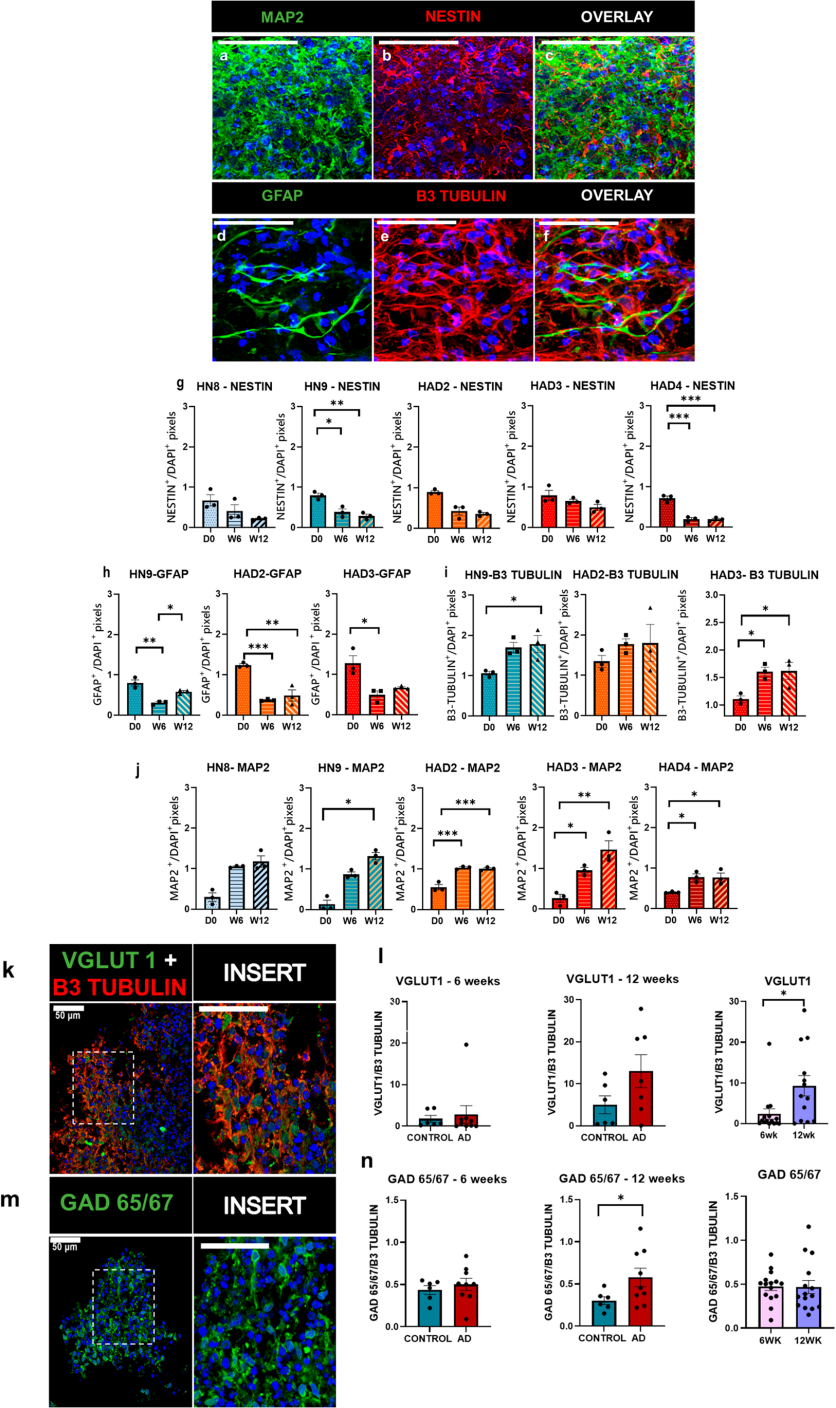
Immunohistochemistry and confocal analysis of 3D cultures at day 0, 6 and 12 weeks: Representative confocal images showing 3D cultures express the neuronal marker MAP2 (**a**), stem/progenitor cell marker Nestin (**b**), astrocytic marker GFAP (**d**) and neuronal marker β3-Tubulin (**e**), glutamatergic marker VGLUT1 (**k**) with β3-Tubulin for better visualisation of the layout of neurons and GABAergic marker GAD65/67 (**m**) at 6 weeks post differentiation. **c** and **f** are overlay of images **a** and **b** and **d** and **e,** respectively. Nestin and β3-Tubulin in red, MAP2, GFAP, VGLUT1 and GAD 65/67 in green, DAPI in blue, scale bar, 50* µm*. Images were taken using 1.3NA × 63 objective glycerol immersion. Quantification of the proportion of positive pixels for Nestin (**g**), GFAP (**h**), β3-Tubulin (**i**) and MAP2 (**j**) relative to DAPI + pixels and VGLUT1and GAD 65/67 ((l and n respectively, left and middle graphs, control vs AD lines; right graphs, all lines combined for 6* weeks* vs 12* weeks*) relative to β3-Tubulin^+^ pixels of individual cell lines at day 0, 6 and 12 weeks post differentiation. *n* = 3 independent culture wells for each cell line at each time point, with 3 images analysed per well. Data represented as mean ± SEM, *p < 0.05, **p < 0.01, ***p < 0.001

The 3D culture-differentiation and cell composition were assessed by immunohistochemistry for neural stem cell/progenitor marker Nestin, neuronal markers β3-Tubulin and MAP2 and astrocytic marker GFAP at different time points (Fig. 2a–f).

Nestin, a type VI intermediate filament protein, is a neural stem cell/progenitor marker, forming a major component of the cell cytoskeleton. Its expression is seen in the cell body and extends to the neurofilaments. There is a gradual decrease in the expression levels of Nestin from day 0 to 6 and 12 weeks post 3D differentiation, significantly, for 3D-HN9 (day 0–6 weeks-*p = 0.0100, day 0–12 weeks-**p = 0.0033) and 3D-HAD4 (day 0–6 weeks-***p = 0.003, day 0–12 weeks-***p = 0.0003), with a similar pattern for 3D-HN8, 3D-HAD2 and 3D-HAD3 (Fig. 2g).

GFAP is localised in the cytoplasmic compartment and is expressed in both astrocytes and stem cell/radial glial. GFAP is expressed as early as day 0 in the 3D cultures. There is a significant decrease from day 0 to 6 and 12 weeks post differentiation in the level of GFAP across cell lines 3D-HN9 (day 0–6 weeks-*p = 0.0011), 3D-HAD2 (day 0–6 weeks-***p = 0.0009, day 0–12 weeks-**p = 0.0020) and 3D-HAD3 (day 0–6 weeks-*p = 0.0219). The expression at day 0 suggests a high proportion of neural stem/progenitor cells, in line with the expression of Nestin. We also observe an increase in GFAP between 6 and 12 weeks for 3D-HN9 (*p = 0.0211), which might indicate an increased proportion of astrocytes (Fig. 2h).

β3-Tubulin, a class III member of the Tubulin family which plays an important role in microtubule assembly, is considered an early neuronal differentiation marker. The majority of cells in 3D cultures express β3-Tubulin as early as day 0. There is a significant increase in the level of β3-Tubulin for 3D-HN9 (day 0–12 weeks-*p = 0.0375) and 3D-HAD3 at 6 (*p = 0.045) and 12-week (*p = 0.0414) post differentiation when compared to day 0 (Fig. 2i).

MAP2, a late neuronal differentiation marker, is expressed in the somatodendritic compartment of neurons, and is seen as early as day 0 in culture and its expression gradually increases to 12 weeks in culture. There is a significant increase in MAP2 levels in the 3D cultures from day 0 to 12 weeks for 3D-HN9 (*p = 0.0219) and from day 0 to 6 and 12 weeks for 3D-HAD2 (day 0–6 weeks-***p = 0.0007, day 0–12 weeks-***p = 0.0009), 3D-HAD3 (day 0–6 weeks-*p = 0.0433, day 0–12 weeks-**p = 0.0031), 3D-HAD4 (day 0–6 weeks-*p = 0.0430), day 0–12 week-*p = 0.0389), with a similar pattern for 3D-HN8. Both the control and AD cell lines show increased levels of MAP2 expression with differentiation time as expected (Fig. 2j).

Synapsin1, a protein found in the pre-synaptic vesicle, is expressed in most of the cells after 6 weeks of differentiation in the 3D cultures (Online Resource 1). VGLUT1, a glutamate transporter protein, is a protein associated with the membranes of synaptic vesicles and is found in the somatodendritic compartment as well as the axonal terminals of excitatory neurons. It is expressed as early as 6 weeks post differentiation in the 3D cultures (Fig. 2k). There is no significant difference between control and AD lines at 6 weeks post differentiation. However, there seems to be a difference, although not significant, at 12 weeks post-differentiation. On analysing the relative proportion of VGLUT 1 in the 3D cultures as a whole (combining all cell lines) over time points 6 and 12 weeks there is a significant increase at 12 weeks compared to 6 weeks of differentiation (*p = 0.0170) (Fig. 2l).

Glutamic acid decarboxylase (GAD) 65/67 synthesises GABA from glutamate and is a marker of inhibitory neurons. The 3D cultures express GAD65/67 as early as 6 weeks post differentiation (Fig. 2m). There are no significant differences between control and AD cultures at 6 weeks post differentiation or between time points 6 and 12 weeks (all cell lines combined) in the differentiated 3D cultures. However, there is a significant difference between the control and AD cultures at 12 weeks post differentiation in the 3D cultures (*p* *0.0409) (Fig. 2n).

In conclusion, the 3D cultures show neuronal and astrocytic differentiation across time, with presence of both glutamatergic and GABAergic neurons, and the expression of synaptic proteins.

### Neuronal differentiation is increased in 3D cultures compared to 2D cultures

**Fig. 3:**
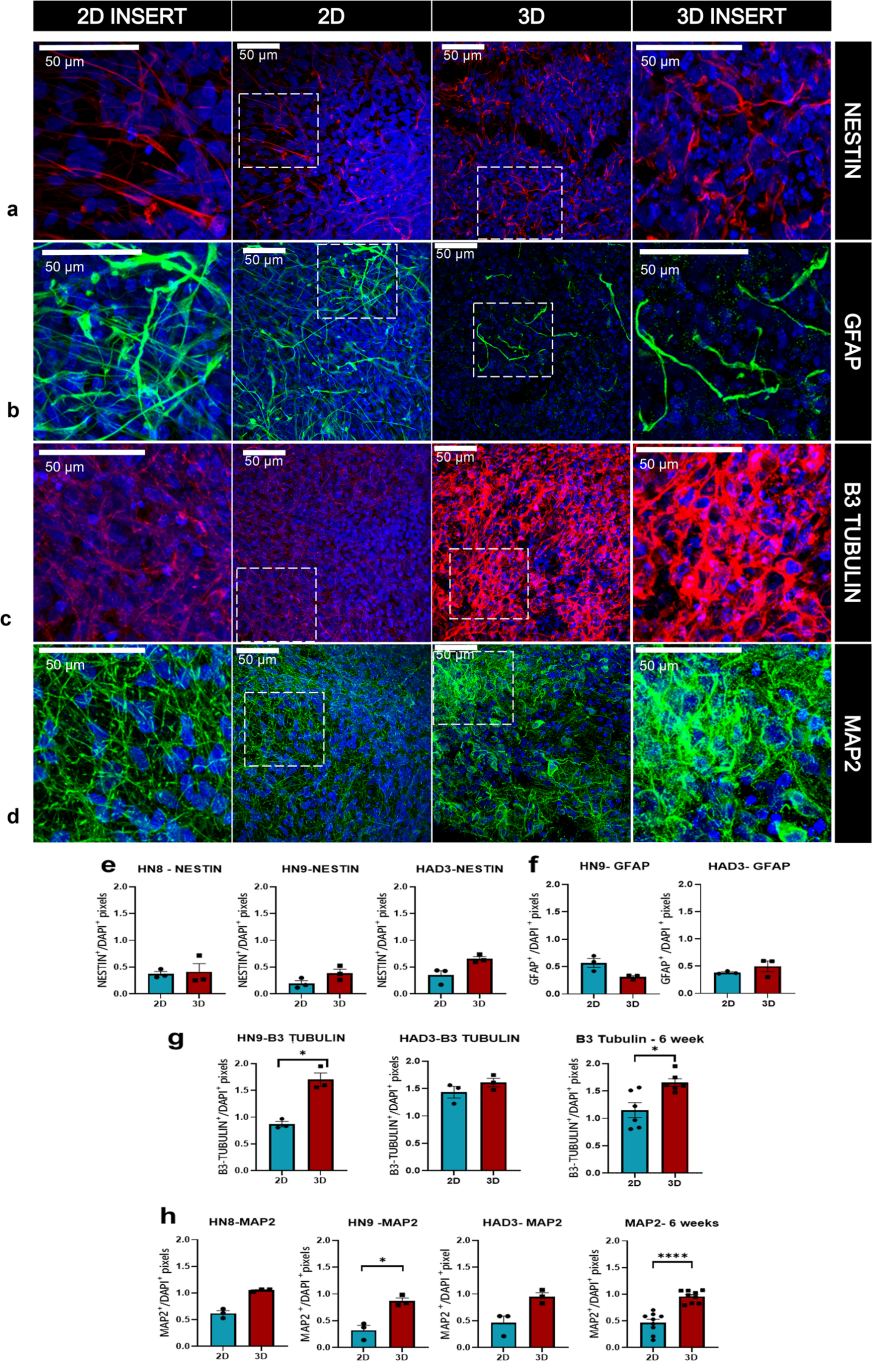
Neuronal differentiation is increased in 3D cultures compared to 2D cultures: representative confocal images of immunohistochemistry of 2D-HAD2 cultures stained for Nestin (**a**), GFAP (**b**), β3-Tubulin (**c**) and MAP2 (**d**). Nestin and β3-Tubulin in red, MAP2 and GFAP in green, DAPI in blue, scale bar, 50* µm*. Images were taken using 1.3NA × 63 objective glycerol immersion. The proportion of Nestin (**e**), GFAP (**f**), β3-Tubulin (**g**) and MAP2 (**h**) positive pixels relative to DAPI + pixels in confocal images across cell lines grown in 2D and 3D culture at 6 weeks post differentiation. **g** and **h** left and middle graphs, data per cell line; right graphs, all lines combined. *n* = 3 independent culture wells for each cell line at each time point, with 3 images analysed per well. Data represented as mean ± SEM, *p < 0.05, ^****^p < 0.0001

It is well known that 2D cultures fail to mimic the *in vivo* microenvironment and thus are not the best candidates for developmental and disease modelling studies. Over recent times, extensive studies have established that 3D cultures are superior to the conventional 2D cultures not just with regards to differentiation and maturation of different cell types but also in the expression of pathological phenotypes *in vitro* [21, 23, 25, 30]. Here, we use 2D cultures in parallel with the 3D cultures for the initial phase of differentiation (day 0–6 weeks) to confirm enhanced neuronal differentiation in our model. The relative expression of the different markers was analysed across culture types (2D and 3D) at 6 weeks post differentiation for different markers: Nestin, GFAP, β3-Tubulin and MAP2 (Fig. 3a–d).

There is no significant difference observed in the levels of Nestin when cells are grown in either 2D or 3D for HN8, HN9 and HAD3 lines (Fig. 3e). Similarly, there was no statistical difference observed for GFAP when grown in 3D vs 2D for the cell lines HN9 and HAD3 (Fig. 3f). We observed that the levels of β3-Tubulin are higher in 3D cultures compared to 2D cultures, statistically significantly for HN9 (*p = 0.0126). However, when the levels of β3-Tubulin in 2D cultures were compared with that of the 3D cultures (cell lines combined) at 6 weeks post differentiation, there was a significant increase seen in the 3D cultures (*p = 0.0122) (Fig. 3g). Similarly, there is a higher proportion of MAP2 in the 3D cultures as compared to the 2D cultures, statistically significantly for HN9 cell line (*p = 0.0116) with a similar pattern for HN8 and HAD3 cell lines. However, when the relative proportion of MAP2 in the 2D cultures was compared to the 3D cultures (cell lines combined) at 6 weeks post differentiation, there was a significantly higher proportion of MAP2 in the 3D cultures (****p < 0.001)(Fig. 3h).

We observed a higher proportion of neuronal markers β3-Tubulin and MAP2 in 3D as compared to 2D, suggesting an increase in the proportion of differentiated neurons in the 3D cultures.

### Expression of total and phospho Tau in the 3D cultures

Although FAD is driven by mutations in APP and PSEN genes, the wild-type Tau becomes abnormally hyperphosphorylated and aggregates during the course of the disease. However, SAD which is not driven by such known mutations and accounts for more than 90% of AD cases also shows similar pathology and clinical presentations to the FAD cases. Despite decades of investigations, the exact cause and the mechanism(s) by which FAD mutations lead to Tau pathology are not yet clear. Here, we addressed this in our FAD 3D culture model and ascertained whether the 3D microenvironment encourages any early changes in the pathophysiological levels of total Tau, especially in its phosphorylation and solubility. We were also interested in studying the expression of 3R and 4R Tau isoforms to determine the predominant maturity of the neurons in the 3D cultures. The adult human brain consists of 6 Tau isoforms, classified as 3R or 4R depending on the presence of 3-repeat or 4-repeat domains at the carboxy-terminal end. The expression of these isoforms is developmentally regulated. 3R Tau has predominantly expressed during the foetal stages and 4R Tau is the adult-specific isoform. In healthy human brains, 3R and 4R Tau isoforms are expressed in equimolar proportions.

**Fig. 4:**
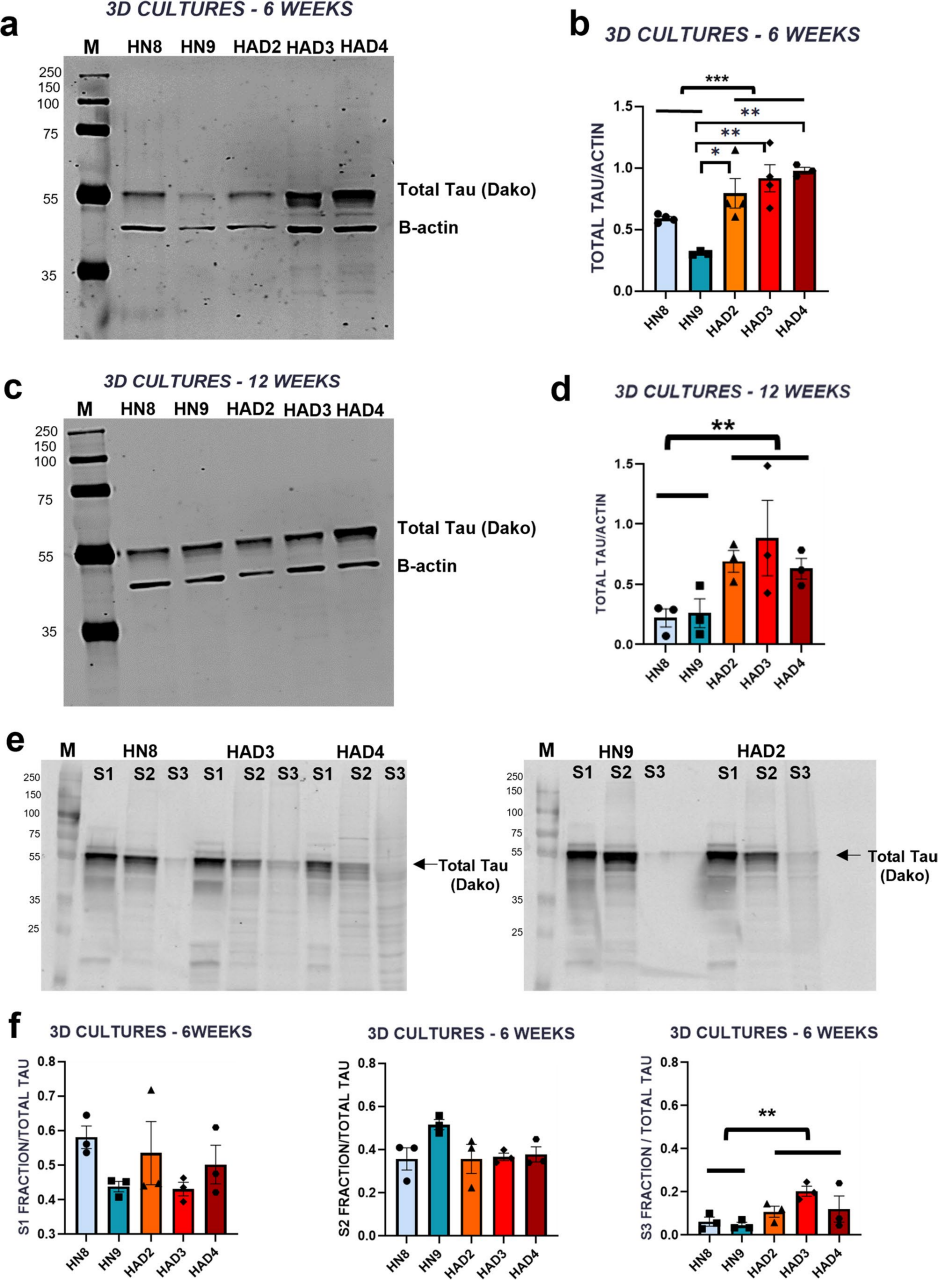
Tau expression and insolubility is higher in 3D AD cells versus 3D control cells: representative western blot images of total lysates of both control and AD -3D cultures at 6 weeks (**a**) and 12 weeks (**c**) post differentiation. The blots were probed with Dako Tau (55 kDa) and β-Actin (42 kDa). Analysis of the western blot data at 6 weeks (**b**) and 12 weeks (**d**) post differentiation. Representative western blot of the fractionated lysates of 3D cultures at 6 weeks post differentiation (**e**). Blots were stained with Dako Tau (55 kDa). Western blot analysis of fractionated protein lysates S1, S2 and S3 fractions from control and AD lines grown in 3D cultures at 6 weeks post differentiation across cell lines (**f**). Equal volumes of normalised samples were loaded in each well. Each fraction is measured as a ratio over total Tau as the sum of all fractions. M, molecular weight marker, *n* = 3 independent culture wells for each cell line at 6 weeks. No statistically significant differences found across individual cell lines grown in 3D cultures at 6 weeks post differentiation. When S1, S2 and S3 fractions from control and AD cells lines were grouped for comparative analysis, there was a significant increase in the proportion of insoluble Tau in the S3 fractions of 3D-AD groups. Data represented as mean ± SEM *p < 0.05, **p < 0.01. ***p < 0.001

To analyse Tau in our 3D cultures, the protein lysates (6 weeks and 12 weeks) were analysed for expression of total Tau by western blotting (Fig. 4). At 6 weeks post differentiation both the controls and AD cell lines express total Tau (Fig. 4a). There are significant differences between the control cell line HN9 and the AD lines HAD2 (*p = 0.0148), HAD3 (**p = 0.0023) and HAD4 (**p = 0.0018) presenting higher levels of total Tau compared to control. However, there is significantly higher total Tau in the AD lines (combined) when compared to control line at 6 weeks post differentiation (***p = 0.0001) (Fig. 4b). There is a trend of the same pattern of increased total Tau levels in AD lines compared to control lines is evident at 12 weeks (Fig. 4c) however, no significant differences are observed at this time point between the different cell lines, although there seems to be a significant increase in the AD lines (combined) when compared to the control lines (combined) (**p = 0.0015) (Fig. 4d). When comparing combined data, there is increased total Tau expression in the AD cell lines compared to the control cell lines, both at 6 and 12 weeks.

Overall, there is an increase in total Tau levels in the AD cell lines compared to the control cell lines, grown in 3D cultures at both 6 and 12 weeks time points. Whether this is a definite or a more transitional change should be assessed further by increasing the number of cell lines and analysing cultures beyond the 12 weeks time point.

Phosphorylation of Tau is considered an important aspect of Tau-induced toxicity in the disease progression. Thus, it was crucial to check if there were any differences between the control and AD lines at different time points. PHF1(Ser 396/404) is an AD-associated phosphorylation epitope. PHF1 is seen in both the controls and the AD-3D cultures at 6 and 12 weeks (Online Resource 2a, c) post differentiation. There was no statistical difference for PHF1 levels between the AD and the control lines at 6 and 12 weeks (Online Resource 2b, d) post-differentiation.

### The level of insoluble Tau is higher in 3D-AD vs 3D-control cells

In AD brains, Tau undergoes conformational changes to transition from soluble monomers to increasingly insoluble oligomers and fibrils, that finally coalesce to form insoluble aggregates. In this experiment, soluble and insoluble Tau levels were assessed by enriching for insoluble Tau fractions obtained by differential centrifugation following standardised techniques [34, 35]. The TBS soluble fraction (S1) shows the presence of monomeric Tau. The TBS insoluble/SDS soluble fraction (S2) shows the presence of Tau oligomers. The Urea soluble S3 fraction shows the presence of insoluble aggregated Tau. In order to check the proportion of Tau in each of the three fractions, lysates of cell lines were fractionated and normalised based on their total protein concentration estimated with BCA assay. Briefly, each fraction was calculated as a ratio of the fraction-band intensity over total Tau normalised against β-Actin (from total lysates). Total Tau was calculated from combining all the fractions S1, S2 and S3.

We observed Tau to be sequestered mostly in the S1 and S2 fractions whereas only a small proportion was found in the S3 fraction across all the cell lines at 6 weeks post differentiation (Fig. 4e). This shows that most of the Tau are soluble in all these cell lines. When we compared the proportion of Tau for each individual cell line grown in 3D culture at 6-week time point, there was no significant difference in any of the fractions across cell lines, though there was a trend for the levels of insoluble Tau in the AD lines to be greater than that seen in controls. When control and AD lines were grouped for cumulative comparison (control vs AD groups), there was a significant increase in the insoluble S3 fraction in the 3D AD group compared to the 3D control group (*p = 0.0078) (Fig. 4f) with no difference in the soluble S1 or S2 fractions between the groups at 6 weeks post differentiation.

Next, we analysed whether there was a difference in the levels of 3R and 4R Tau isoforms between control and AD cell lines. We observed 3R Tau was mostly found in the S1 and the S2 fractions in all the cell lines and a very small proportion in the insoluble S3 fraction of the 3D cultures at 6 weeks post differentiation (Online Resource 3a). There was no significant difference in the proportion of 3R Tau in the three fractions across the cell lines at 6 weeks post differentiation (Online Resource 3 b-d). Interestingly, when control and AD lines were grouped for cumulative analysis (control vs AD groups), there was a significant decrease in the proportion of 3R Tau in the S2 fraction (*p = 0.0310) and a significant increase in the S3 fraction (*p = 0.0176) from 3D-AD cultures when collectively compared to the 3D control lines (Online resource 3c-d). The adult 4R Tau isoforms were detected at 6 weeks in both control and AD 3D cultures, however, 4R Tau bands were too low in intensity to quantify (Online resource 3e).

Thus, we have generated a human 3D-AD neural culture model which shows changes in the solubility of Tau and the presence of aggregated Tau as early as 6 weeks post differentiation. Although at 6 weeks the proportion of urea insoluble vs soluble Tau in the 3D cultures is quite low, there is a trend for more detergent insoluble Tau in the AD lines when compared to the healthy control lines. Though these trends were only significant when the AD and controls lines were grouped for cumulative analysis; future studies should assess if this difference is evident with a greater number of cell lines without grouping.

## Discussion

Development of a human *in vitro* model for a neurodegenerative disease like AD which replicates most if not all pathological hallmarks is quite challenging. Some studies have previously reported AD phenotypes in their 3D/organoid cultures using either immortal neural cell lines or by forcing the pathology by various means [23, 25, 36]. Although these models recapitulate some of the AD biochemical phenotypes and constituted seminal works at the time, they also lack the relevant pathophysiological levels of protein expression. In this study, we address this shortcoming by using iPSCs derived from FAD patients, thereby, bypassing the need to overexpress or genetically manipulate any FAD genes, allowing us to study the disease without overexpressing mutated genes. 3D cultures are great tools to recreate a physiologically relevant brain microenvironment and significantly improve the maturation and functionality of the different neuronal cell types [37–39]. Here, we have developed a human 3D neural culture model using iPSC-derived NSC from healthy individuals and FAD patients with PSEN1 mutations-*L286V*,* M146L *or* A246E,* maintained for 12 weeks post-3D plating *in vitro.* Our model shows early signs of neuronal maturation in the 3D cultures, increased total Tau in 3D-AD when compared to the 3D controls, and early signs of Tau insolubility from cells derived from iPSCs from patients with PSEN1 mutations. Protein aggregation being the primary hallmark of a disease like AD, an ideal model would be one that reflects brain-like compartmentalization, which is instrumental to help mimic the relevant microenvironment and recreate the pathology *in vitro*. A 3D culture system could be engineered to integrate a heterogeneous population of cell types dispersed in three dimensions enhancing cell–cell communication in the intracellular niche, embedded with specific scaffold materials, extra-cellular matrix components (ECM) and growth factors. This would not just accelerate maturation but also provide the compartmentalization needed to accumulate pathological phenotype, as shown by other studies [23, 25]. Using Matrigel, which contains a high level of brain ECM proteins such as laminin, entactin, collagen and heparin sulphate proteoglycans, we see a more robust neuronal differentiation in 3D cultures compared to the 2D cultures. The 3D cultures also contain GFAP^+^ astrocytes, with the proportion of neurons and astrocytes increasing while Nestin^+^ progenitor cells decreased over time. Expression of Synapsin1, a pre-synaptic marker is seen as early as 6 weeks post 3D plating, alongside glutamatergic marker VGLUT1 and GABAergic marker GAD65/67. Our heterogeneous human neural 3D culture model shows neuronal maturation and neural network formation as early as 6 weeks post 3D plating. This enhanced differentiation could be attributed to a more physiologically relevant microenvironment and promoting neuronal differentiation in 3D cultures.

The 3D AD cultures increased displayed increased total Tau and some evidence of greater insoluble Tau as compared to the 3D control cultures at 6 weeks post differentiation. This suggests that Tau aggregation in FAD may not be a end-stage process and starts earlier than expected, supporting the thought that AD pathology begins many years before the onset of clinical symptoms. This is a good *in vitro* model to explore protein aggregation and its effects and may be adapted to study other neurodegenerative diseases such as Parkinson’s disease (PD) and Demetia with Lewy bodies (DLB). Tau is abnormally hyperphosphorylated and aggregated in the form of paired helical filaments (PHFs) which progresses to form tangles in AD [29]. There are no significant changes in the PHF1 in 3D-AD lines when compared to 3D controls at 6 weeks and 12 weeks post-differentiation. However, for further analysis, we will try using other phospho-epitopes such as AT8 (Ser 202, Thr 205). A study in 2015 using cortical neurons derived from patient-specific iPSC also reported no change in PHF1 levels in their PSEN 1 (PSEN1 Y115C, M146I, intron 4) as opposed to the APP cell lines used (APP V717I mutant cells). Moreover, this study reported an increase in total Tau only in the APP mutant lines, and not in the PSEN1 lines [33], whereas, we see a total Tau expression increase in PSEN1 lines.

One of the challenges in modelling an age-related neurodegenerative disease like AD is to generate mature and aged neurons. Although publications on 3D-AD culture models have shown some neuronal maturity with different markers, most do not show expression of the adult 4R Tau isoform at the protein level [24, 25, 30]. However, a few 3D/organoid models studies show expression of 4R Tau at the protein level, following long-term cultures of 300 days [40] and 365 days [41]*,* or long-term use of Brain-Phys with expression at 25 weeks [42]. Although, we do not see all six isoforms, we do see 4R isoforms expression as early as 6 weeks after 3D plating. We believe the expression of the 4R isoforms could become more prominent with extended culture times as shown by the above-mentioned studies. This shows that our 3D culture provides an environment for increased differentiation and more efficient maturation and ageing of the neurons.

Premature neuronal differentiation might occur early during development in FAD patients with PSEN1 mutations both *in vitro* and *in vivo* [43, 44]. This might explain the early pathogenic differences seen between the control and AD 3D cultures.

Studies have shown that neuronal ageing is challenging to replicate *in vitro* as reprogrammed iPSCs usually differentiate into immature or foetal-like neurons which are quite different from those at the age of onset of diseases like AD [45, 46]. Although their maturity may be far from the age of onset, these immature cells display pathological attributes which support the concept that pathological changes happen in the pre-symptomatic phase of the disease, years before age of onset, and that such models are great tools to study and target early changes in diseases. However, an in-depth characterisation of the different cell types and proportions in our 3D cultures needs to be done in the future to completely understand the extent and maturity of cortical differentiation. This also includes a clear distinction between the type of glial cells. Although GFAP is a classic astrocytic marker, it is a marker also expressed in the radial glial cells. Currently, there is no single marker that could distinguish between early and late astrocytes due to a general overlap seen with the available markers. A combination of markers such as aquaporin 4 (AQPR 4) or S100β with GFAP could be used to get a clear characterisation of the cell type [47, 48]. The accurate sub-cellular localization of the markers can also give an indication of the physiological conditions. While GFAP is found in the cell processes, S100β is found in the soma of mature astrocytes [49, 50] and AQP4 is found in the end feet of astrocytes [51, 52].

Although there is a rapid development in the field of neural 3D/organoid cultures, they do come with their own limitations [20]. Some of these limitations are the absence of crucial elements which make the brain a complex organ. Lack of microglial compartment, vasculature and blood–brain barrier separates us from the goal towards a physiologically relevant model. In recent years, the advances in microfluidic systems have made it possible to address these issues [53]. A human 3D triculture microfluidic system was used to demonstrate simultaneous culture of neuron, astrocytes and microglial capturing AD features such as Aβ aggregation, accumulation of phosphorylated Tau, neuroinflammation and microglial recruitment [54]. Although an immortal microglial cell line was used, studies like these promise a platform to work with patient-specific isogenic lines. Introducing elements of vasculature and blood–brain barrier would be advantageous in reducing necrotic cores, a long-standing problem seen in organoid cultures [55].

3D culture systems are widely applicable wherein they can be tailored not just for disease modelling or drug targeting studies but also to help uncover numerous unknown physiological and pathological mechanisms. For instance, recent studies have led to believe reduced expression of perivascular AQP4, a major regulator of the glymphatic system affects the clearance of toxic solutes such as Aβ [56–61]. 3D models could be employed to further study clearance mechanisms in the brain and pave way for developing effective drugs for diseases like AD.

The field of 3D/organoid/spheroid culture technology is constantly striving to make the system more adaptable for advanced imaging techniques such as TEM, expansion microscopy as well as live functional readouts such as calcium imaging and multielectrode array (MEA) to study neuronal connections and activity and thereby overcome some limitations in the field [62–64]. These adaptations would help study effects in real-time to explore mechanisms such as neuroinflammation, microglial activation and blood–brain barrier using various advanced platforms such as microfluidics, microvessel-on-a-chip and Organ-on-Chip technology [65, 66]. Further advances in technology such as 3D-bioprinting are underway to enable a multicellular and multi-organ research approach to improve therapeutic potentials [67].

In conclusion, our human iPSC-derived 3D neural culture model is a promising and physiologically relevant system with a mix of astrocytes and mature neurons, including both excitatory and inhibitory neurons, expressing some features of the mature Tau as early as 6 weeks *in vitro*. We believe that the 3D culture would be a good model to study other aspects of the AD pathology such as amyloid pathology, synaptic abnormalities and neuroinflammation with microglia, and may also be used for compound screening in the future.

## Supplementary Information

Below is the link to the electronic supplementary material.

Supplementary file1 (DOCX 2206 KB)

## Data Availability

Supporting information is provided separately with the article. The datasets generated during and/or analysed during the current study are available from the corresponding author on reasonable request (data will be made freely available in the University of Southampton’s repository after publication).
