# Supplementary material for: A 3D-induced pluripotent stem cell-derived human neural culture model to study certain molecular and biochemical aspects of Alzheimer’s disease
Source: In Vitro Model. 2022 Nov 14;1(6):447–62. doi: 10.1007/s44164-022-00038-5 (PMC11756488; doi:10.1007/s44164-022-00038-5)
Supplement: Supplementary file 1 — Supplementary file1 (DOCX 2206 KB) [file 44164_2022_38_MOESM1_ESM.docx]

# A 3D- Induced Pluripotent Stem Cell-Derived Human Neural Culture Model to Study Certain Molecular and Biochemical Aspects of Alzheimer’s Disease.

Preeti Prasannan^1^

Elodie Siney^1^

Shreyasi Chatterjee^2^

David Johnston^1^

Mohammad Shah^1^

Amrit Mudher^2^

Sandrine Willaime-Morawek^1^

Affiliations:

^1^ Faculty of Medicine, University of Southampton, Southampton, U.K.

^2^ School of Biological Sciences, University of Southampton, Southampton, U.K.

Corresponding Author: Sandrine Willaime-Morawek (ORCID- 0000-0002-1121-6419)

[S.Willaime-Morawek@soton.ac.uk](mailto:S.Willaime-Morawek@soton.ac.uk)

## **Supplementary Figures**

***
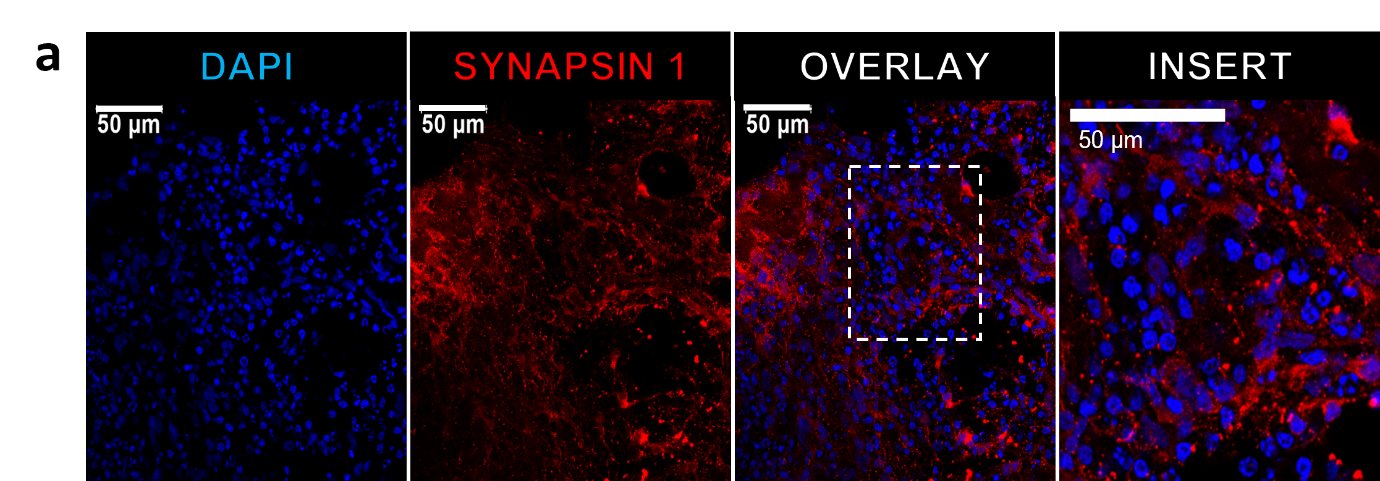
 Online Resource 1 Immunohistochemistry of 3D cultures at 6 weeks*** *Representative confocal images of HAD3 showing 3D cultures express the pre-synaptic marker Synapsin1 (a) at 10 weeks post differentiation. Synapsin 1 in red, DAPI in blue. Images were taken using 1.3NA x63 objective glycerol immersion, scale bar, 50 µm.*


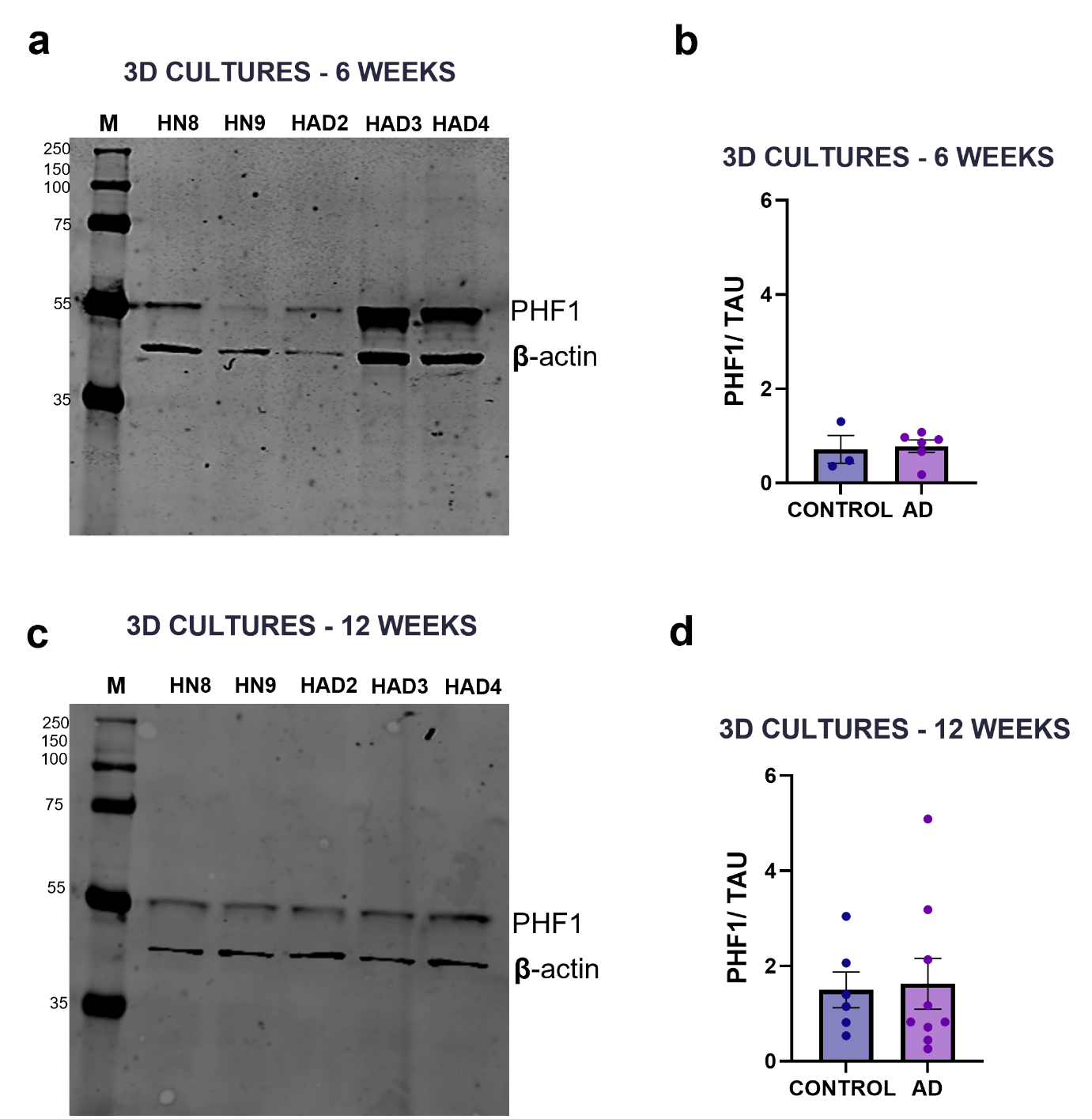


***Online Resource 2 PHF1 phosphorylation of Tau in the 3D cultures*** *Representative western blot images of total lysates of both control and AD -3D cultures at 6 weeks (a) and 12 weeks (c) post differentiation. The blots were probed with PHF1 Tau (55kDA) and β-Actin (42kDA). Each well was loaded with 15µg of protein, M=molecular weight marker. n=3 independent culture wells for each cell line at each timepoint. Quantification of western blot band intensity for PHF1 and β-Actin at 6 weeks (b) and 12 weeks (d) post differentiation. Data represented as mean ± SEM, no statistically significant differences found*

***
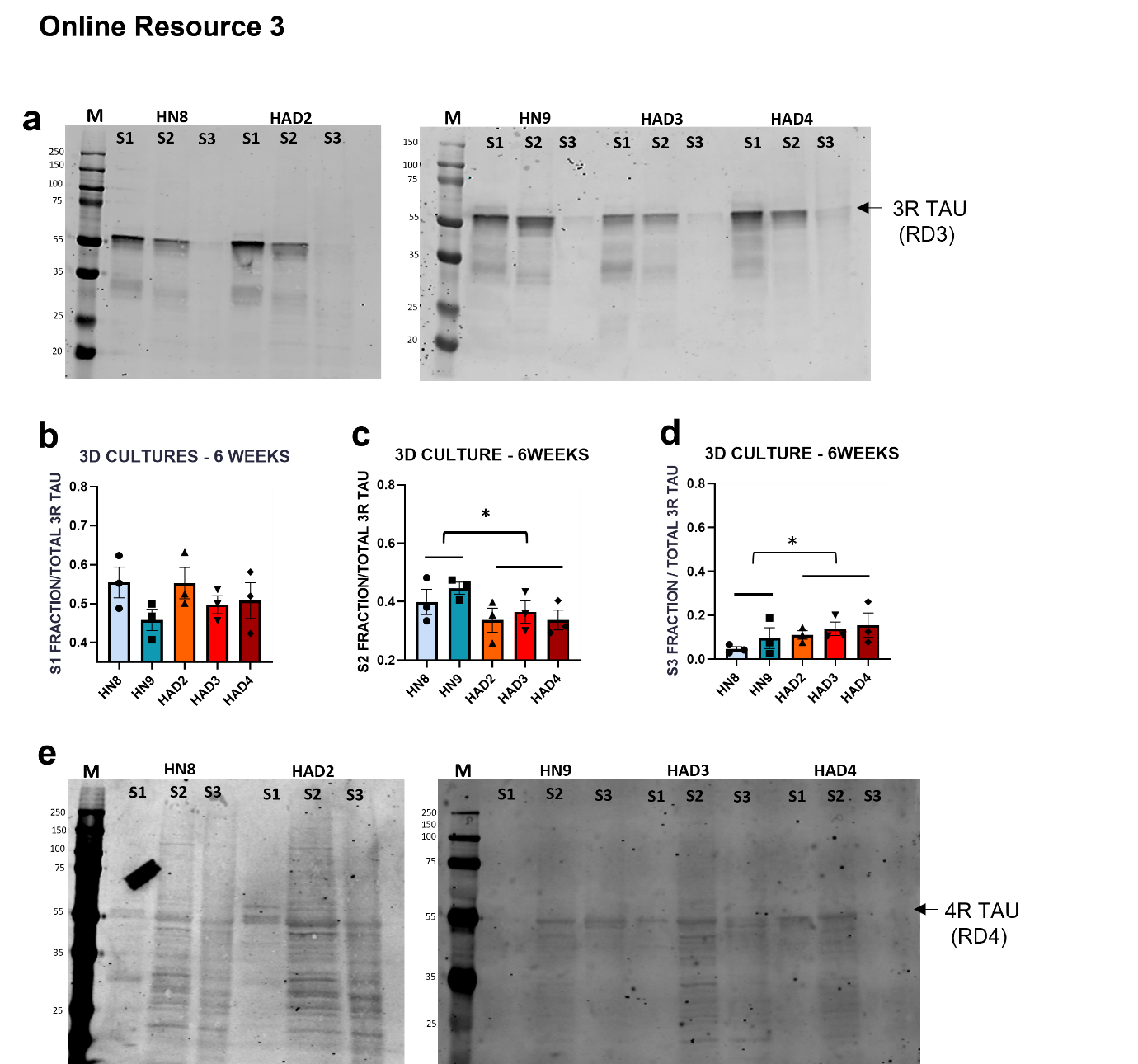
***

***Online Resource 3 Solubility of Tau isoforms in the 3D culture*** *Representative western blot of the fractionated lysates of 3D cultures at 6 weeks post differentiation, stained with 3R Tau (45-65kDa, a). Quantitative western blot analysis of 3R Tau (b, c, d) fractionated lysates of 3D cultures S1, S2 and S3 fractions at 6 weeks post differentiation across cell lines. Equal volumes of normalised samples were loaded in each well. Each fraction is measured as a ratio over Total Tau as the sum of all fractions. No statistically significant differences found across individual cell lines grown in 3D cultures at 6 weeks post differentiation. When S1, S2 and S3 fractions from control and AD cell lines were grouped for comparative analysis, there was a significant decrease in the proportion of Tau in the S2 fraction and a significant increase in the S3 fraction of 3D-AD groups. Representative western blot of the fractionated lysates of 3D cultures at 6 weeks post differentiation, stained with 4R Tau (68-72 kDa, e). M=molecular weight marker, n=3 independent culture wells for each cell line at 6 weeks post differentiation. *p<0.05*
